# Drug-induced tumor-specific cytotoxicity in a whole tissue *ex vivo* model of human pancreatic ductal adenocarcinoma

**DOI:** 10.3389/fonc.2022.965182

**Published:** 2022-08-18

**Authors:** Carlos Fernández Moro, Arun Kumar Selvam, Mehran Ghaderi, Ville N. Pimenoff, Marco Gerling, Béla Bozóky, Soledad Pouso Elduayen, Joakim Dillner, Mikael Björnstedt

**Affiliations:** ^1^ Department of Laboratory Medicine, Division of Pathology F46, Karolinska Institutet, Karolinska University Hospital Huddinge, Stockholm, Sweden; ^2^ Department of Clinical Pathology and Cancer Diagnostics, Karolinska University Hospital, Stockholm, Sweden; ^3^ Department of Biosciences and Nutrition, Karolinska Institutet, Huddinge, Sweden; ^4^ Tema Cancer, Karolinska University Hospital, Stockholm, Sweden

**Keywords:** pancreatic ductal adenocarcinoma, sodium selenite, drug-resistant tumor, pancreatic cancer, *ex vivo* model, tissue slice model, drug testing

## Abstract

Pancreatic ductal adenocarcinoma (PDAC) is the most common type of pancreatic cancer. PDAC has a dismal prognosis and an inherent resistance to cytostatic drugs. The lack of reliable experimental models is a severe limitation for drug development targeting PDAC. We have employed a whole tissue *ex vivo* culture model to explore the effect of redox-modulation by sodium selenite on the viability and growth of PDAC. Drug-resistant tumors are more vulnerable to redox-active selenium compounds because of high metabolic activity and redox imbalance. Sodium selenite efficiently and specifically reduced PDAC cell viability (p <0.02) (n=8) and decreased viable *de novo* tumor cell outgrowth (p<0.05) while preserving non-neoplastic tissues. Major cellular responses (damaged tumor cells > 90%, tumor regression grades III-IV according to Evans) were observed for sodium selenite concentrations between 15-30 µM. Moreover, selenium levels used in this study were significantly below the previously reported maximum tolerated dose for humans. Transcriptome data analysis revealed decreased expression of genes known to drive PDAC growth and metastatic potential (CEMIP, DDR2, PLOD2, P4HA1) while the cell death-inducing genes (ATF3, ACHE) were significantly upregulated (p<0.0001). In conclusion, we report that sodium selenite has an extraordinary efficacy and specificity against drug-resistant pancreatic cancer in an organotypic slice culture model. Our *ex vivo* organotypic tissue slice culture model can be used to test a variety of drug candidates for swift and reliable drug responses to individual PDAC cases.

## Introduction

Pancreatic cancer is currently the fourth leading cause of cancer-related death in the Western world, and it is anticipated to rank second by 2030 (1). Pancreatic ductal adenocarcinoma (PDAC) is the most common form of pancreatic malignancies, with a 5-year survival rate of less than 9% (1, 2). The characteristics of the disease are advanced stage at diagnosis, a high propensity for metastatic spread and remarkable resistance to chemotherapy (3).

Unfortunately, only about 20% of patients are eligible for surgery (4) and in the majority of patients the tumor is locally too advanced or has spread to distant locations, precluding the survival benefit of surgery. Until 2011, monotherapy with gemcitabine remained standard treatment. Thereafter, the ESPAC-4 trial demonstrated a superior 5-year survival for combination treatment with gemcitabine and capecitabine (GemCap, 28.8%) compared to gemcitabine only (16.3%) (5). Recent clinical trials have demonstrated a survival benefit for FOLFIRINOX (folinic acid, fluorouracil, irinotecan and oxaliplatin; 11.1 vs. 8.8 months) (6) or nab-paclitaxel-gemcitabine (8.7 vs 6.6 months) versus gemcitabine alone (7). However, both combination regimens are associated with substantial toxicity, limiting their use to patients with good performance status without relevant comorbidities (7). Regardless of the treatment regime, in most patients the disease progresses within a few months, which illustrates the critical need for more effective treatments both for first- and second-line therapy.

Redox-active selenium compounds are interesting candidates for the treatment of pancreatic cancer given its pronounced drug resistance; it has been shown by our group and others that drug-resistant tumor cells are more sensitive to the cytotoxic effects of selenium compounds compared to non-malignant cells (8). The tumor-specific cytotoxicity of selenium compounds can be attributed to the altered redox status of cancer cells (9, 10), due to elevated intra- and extracellular thiol levels, which in several instances infers drug resistance (11). Malignant cells can upregulate intracellular redox buffer systems, e.g glutathione (GSH), to evade increased cellular stress, *i.e.*, reactive oxygen species (ROS) production triggered by high proliferation rates (12). To maintain a high capacity of the redox systems, a continuous supply of cysteine is required which is ensured by the cystine/glutamate (xCT) antiporter, generally upregulated in resistant malignant cells. The xCT antiporter is also crucial for selenium uptake after sodium selenite administration, a mechanism that can explain the striking tumor specificity of sodium selenite (11). A leading mode of action of selenium cytotoxicity is the induction of oxidative stress arising from redox cycles of low-molecular-weight selenolates with thiols and oxygen (13). These cycles, maintained by GSH or the thioredoxin system, are very efficient and may produce high levels of ROS non-stoichiometrically (14). We have previously shown in a Phase-I academic clinical trial that sodium selenite is safely tolerable in humans. It has a maximum tolerated dose of 10.2 mg (Se)/square meter body surface, which is well above the doses used in the present study (15).

Sodium selenite exerts its cytotoxic effects by directly oxidizing cellular free thiols (16). Selenium is readily taken up by pancreatic and liver cells upon oral administration of ^75^Se (17). Thereof, selenium compounds have been proposed as potentially highly effective drug candidates for the treatment of pancreatic cancer.

The choice of the experimental model plays a crucial role in the investigation of selenium-mediated cancer cell cytotoxicity. The conventional approach for drug testing involves the use of commercially available, immortalized cells (18). Such cell lines are readily accessible and easy to handle for high-throughput drug screening. The limitations of cell line-based systems include loss of phenotypic characteristics specific to the original malignancy and lack of the tumor microenvironment. Furthermore, animal models in cancer research are mainly xenograft mouse models transplanted with human tumor cells, and genetically engineered mouse models (19). However, interspecies discrepancies render these results difficult to translate to humans. Developing new experimental models that closely resemble a human *in vivo* setting is of utmost importance to overcome these limitations. Hence, we have recently established a novel *ex vivo* organotypic culture system where precision-cut slices of human PDAC tissue obtained from surgical specimens are cultured with good preservation of tissue integrity and viability, allowing drug sensitivity testing of an individual patient’s cancer (20).

Here, we use the *ex vivo* tissue slice model in human PDAC specimens for drug sensitivity testing and demonstrate, for the first time, a selective antitumor effect of sodium selenite on PDAC cell viability. The effect was observed at dose levels well tolerated by humans, while non-neoplastic tissue was shown unaffected.

## Materials and methods

### Patients and tissue samples

Fresh tumor tissue samples were collected from surgical specimens of primary PDAC (n=8 individual patients; culture IDs DT1-DT8; DT referring to “organotypic drug testing”) resected at Karolinska University Hospital between January and November 2020. Clinicopathological characteristics are presented in **Table 1**.

**Table 1:** Clinicopathological data.

Culture ID	Gender	Preoperative chemotherapy	Histological type	Grade of differentiation	Stage*
**DT1**	Female	No	PDAC	Moderate	pT3 N2
**DT2**	Male	No	PDAC	Poor	pT2 N1
**DT3**	Male	No	PDAC	Moderate	pT2 N1
**DT4**	Male	No	PDAC	Moderate	PT3 N0
**DT5**	Female	No	PDAC	Moderate	pT3 N2 M1
**DT6**	Male	No	PDAC	Poor	pT3 N2 M1
**DT7**	Male	No	PDAC	Moderate	pT3 N2
**DT8**	Female	No	PDAC	Moderate-poor	pT3 N2

*Stage – TNM classification (8th Edition).

The study was approved by the Regional Ethical Review Board, Stockholm/Etikprövningsmyndigheten (decision numbers 2012/1657-31/4, 2018-2654/32 and 2019-00788). Written informed consent was obtained from all patients before surgery. All study procedures were performed following the relevant guidelines and regulations.

### Preparation of precision-cut tissue slices

**Figure 1:**
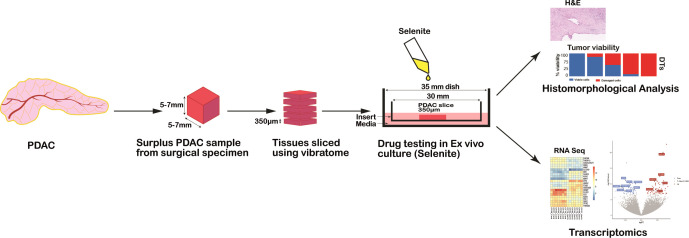
Schematic representation of the workflow for drug testing in PDAC *ex vivo* model from human surgical specimens.

A piece of tumor tissue (approximate dimensions: 6-10 mm long, 5 mm width and 5-7 mm height) was cut freshly using a vibrating-blade vibratome (VT1200S, Leica, Germany) into 350 μm thick slices according to the method established before by our group (20) (**Figure 1**).

The slicing procedure yielded on average 14 slices (range: 9-18) per tumor sample. The slice order was recorded by placing the slides consecutively in a 24-well plate containing ice-cold transport medium. Whenever possible, larger slices were halved with small surgical scissors to increase the total number of slices for culture.

### Slice culture and treatment conditions

Slices were cultured for a total of 72h at atmospheric oxygen level (21% O_2_) rested on an insert (0.4 µm pore size, Millicell^®^, Millipore, Ireland) placed in a culture dish containing complete culture media, as previously described (20) except the addition of diphenyl diselenide. After 24h, slices were treated during subsequent 48h of culture by replacing the culture medium with a fresh medium spiked with the following drugs and concentrations: sodium selenite (Se, Sigma-Aldrich, Darmstadt, Germany) at 5 µM, 15 µM and 30 µM, and gemcitabine at 1 µM concentration. Untreated cultured slices were used as controls (referred to as Control 72h). The different treatment conditions were tested in duplicate slices.

### Qualitative histomorphological assessment

After culture, slices were formalin-fixed, embedded in paraffin and processed for histology. To avoid loss of tissue with multiple sectioning procedures, tissue sections were obtained up-front for hematoxylin-eosin (H&E) (3 x 4 µm sections), transcriptome analysis (1 x 10 µm) and unstained slides for immunohistochemistry (5 x 4 µm sections). The latter were stored at 4°C until processing for analysis. H&E sections were evaluated by a specialist pancreatic pathologist (CFM) to confirm the presence of PDAC and to assess the grade of tumor differentiation.

### Immunohistochemistry

Cytokeratin (CK) 19 immunohistochemical staining (clone b170, concentration 1:100, product code NCL-CK19, manufacturer Novocastra Leica BiosystemsLtd, Newcastle Upon Tyne, United Kingdom) was used to demarcate the tumor regions in one culture (DT2) that showed extensive infiltrate of a poorly differentiated adenocarcinoma. Staining was performed using a Leica BOND III automated immunostainer.

### Digital histological slides

H&E (DT1-DT8) and immunohistochemically (DT2) stained slides were digitalized using a Hamamatsu NanoZoomer slide scanner at 20X magnification. Histomorphological quantitations were performed on the whole slide images using QuPath v0.2 (21).

### Readouts for the assessment of drug response-associated cell damage in the tissue slices: tumor viability, cancerous cell outgrowth length and tumor viability index

Our previous study on precision-cut slices of human PDAC revealed the gradual appearance of a cancerous cell outgrowth during culture on the surface of the tissue slices (20). Following preliminary explorative investigations, we considered as most reliable readouts of tumor cell viability and response to treatment: the percentage of viable vs severely damaged tumor, either in cancerous cell outgrowth (n=6) or within the slice (n=2); the percentage length of cancerous cell outgrowth with respect to the slice perimeter (n=6); and a cancer cell viability index (n=6), calculated as the summation of the products of the outgrowth lengths and viability weights, according to the formula “Σ_i=viable, damaged_ (% outgrowth length_i_ * weight_i_)”, where weight equals 3 and 1 for viable and severely damaged cell outgrowth, respectively. Tumor viability was assessed within the slice in two cultures that did not develop cell outgrowth, corresponding to a poorly differentiated carcinoma (DT2) and a moderately differentiated carcinoma that showed dispersed, insidious type of invasion between abundant regions of pancreatic parenchyma in a background of chronic pancreatitis (DT4).

The cancerous cell outgrowth was annotated manually on H&E-stained whole slide images (n=94) by a specialized pancreatic pathologist (CFM), according to its different morphological appearances (see below): flat, cubic, cylindrical, clear, swollen and necro-apoptotic. The perimeter of the tissue slice was also annotated.

Similarly, viable and severely damaged tumor regions were annotated within the slice on whole slide images (n=35) for the two cultures described above, and their relative percentages were calculated for each slice. For one culture with an insidious growth pattern intermixed with remnants of non-tumorous pancreatic parenchyma (DT4), the regions of viable and severely damaged tumor cells were manually annotated on H&E-stained slides. While for a poorly differentiated carcinoma (DT2), the total tumor area was delineated based on CK19 staining using QuPath’s pixel classifier and then the limited areas of severely damaged (in control, untreated slices) or viable (in the treated ones) tumor were manually annotated.

### Data processing

Quantitative data derived from the annotations (lengths in µm and areas in µm^2^) were exported from QuPath in tabular format and processed using statistical software, R v3.6.3 (22). Briefly, for outgrowth forming tumors (n=6), the percentage lengths of the different morphological cell outgrowths for the slice perimeter were calculated. While for the other two tumors, the respective percentages of viable and severely damaged tumor areas within the slice were calculated. Values were then averaged over duplicate slices for each culture and condition. Subsequently, the percentages of cell outgrowths were grouped into “viable” (comprising flat, cubic, cylindrical, and clear) and severely “damaged” (comprising swollen and necro-apoptotic) categories and their respective percentages were calculated.

### Isolation of total-RNA and library preparation

Seven out of eight tumors were sequenced, comprising five moderately differentiated and two poorly differentiated carcinomas. DNase-treated total RNA was extracted from FFPE sections using the Maxwell RSC FFPE RNA kit (Promega, Madison, USA). Extracted RNA was quantified by Qubit 4.0 using the RNA HS Assay Kit (Thermofisher Scientific, Waltham, USA). A maximum of fifty nanograms of extracted RNA was used to prepare cDNA libraries after fragmentation of total RNA at 94°C for 3 minutes. Whole transcriptome sequencing libraries were prepared using the Takara Smarter total-RNA Seq kit V2.5 Pico Input Mammalian (Takara Bio Inc, Kusato, Japan). Briefly, cDNA libraries were prepared by modified random hexamer priming oligos. During a first PCR amplification, full-length Illumina adapters, including barcodes were added. The ribosomal cDNA sequences (originating from rRNA) were depleted in the presence of RNAse H and the mammalian-specific R-Probes. The remaining fragments were enriched *via* a second round of PCR amplification using primers universal to all adapters. The final library contained sequences allowing clustering on any Illumina flow cell. To equalize the amount of the input from each sample, cDNA libraries were quantified by Qubit 4.0 HS Assay Kit (Thermofisher Scientific, Waltham, USA).

### NextSeq 500 sequencing, bioinformatic analysis and statistical evaluation

All samples were sequenced on a NextSeq 500 Illumina system (Illumina, San Diego, USA). Paired-end cycle sequencing 2×75 was run on mid-output V2.5 Kit which in total generated a median of 25 million raw paired-end reads/sample. Takara indices, according to TruSeq 96 CD Illumina adapters, were used to demultiplex and assign raw sequence reads. Datasets were analyzed using bioinformatic tools at Chipster virtual interface at CSC Finland (23) to process and analyze RNA data for gene expression. Adapters were preprocessed and trimmed thereby all sequences were quality-checked by FastQC. Paired-end reads were mapped using STAR aligner on Homo sapiens genome version release GRCh38.95 (24). Quantitation of sequencing reads in BAM files for each gene was estimated by HTSeq which resulted in the aligned read counts per all sequenced gene transcripts (25). Differential expression analysis was performed using the DESeq2 Bioconductor package. In brief, normalized control and treatment count tables were merged into one and were used as a template for differential expression analysis and to generate fold change values in the log2 scale. Genes with an adjusted p-value ≤ 0.05 and log2fold change of +1 or -1 were initially considered as significantly expressed. To evaluate the most significant differentially expressed genes, sequencing data were filtered by restricting the p-value to ≤ 0.0001 following the exclusion of transcripts with very low, less than 350 reads, alignment abundancy. Hierarchical clustering heatmaps and dendrograms of RNA expression profiles were generated using DESeq2 software package. Volcano plots were drawn using the open-source Galaxy platform (26). Pathway analysis of all differentially expressed genes was done on the “Reactome database” (27).

### Statistical analysis

The proportion of viable and severely damaged tumor cells in outgrowth and within the slice was calculated for the readout tumor viability (n=8). The total percentage of cancer cell outgrowth (viable and severely damaged cells combined) was also calculated for the readout cancerous outgrowth length (n=6). The percentage of cancer cell outgrowth multiplied by viability weights was used for the readout tumor viability index. Shapiro-Wilk test was used to indicate the non-normal distribution of the tumor viability (p< 0.001), tumor outgrowth length (p= 0.014) and tumor cell outgrowth viability index (p< 0.001) data. Non-parametric Wilcoxon signed-rank test was used for the paired comparisons between control (72h cultured, untreated) and the different treatment conditions with Benjamini-Hochberg correction for multiple testing. In addition, to estimate the global difference across the multiple treatment conditions a Friendman test was applied. An adjusted p-value of < 0.05 was considered statistically significant.

## Results

### Histological evaluation revealed distinct cancer cell morphologies associated with different grades of tumor cell damage and viability in organotypic tissue slices

**Figure 2:**
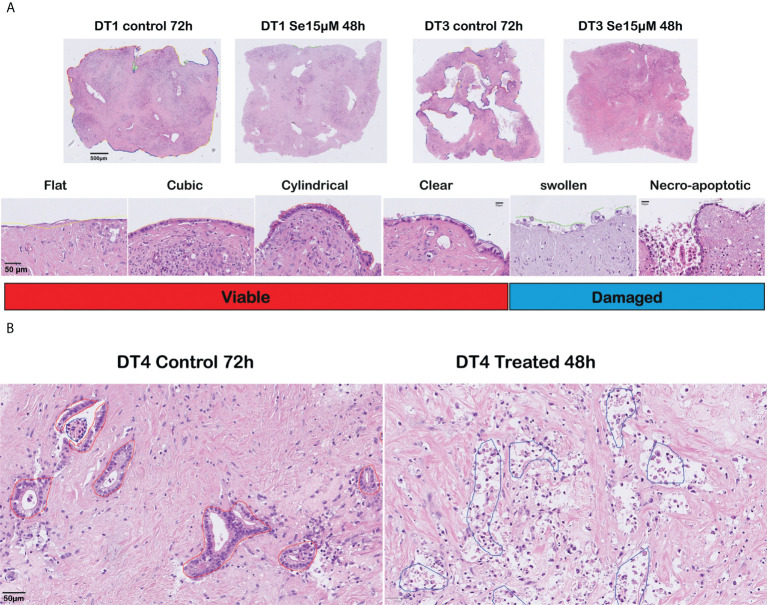
Histomorphological assessment of responses to sodium selenite treatments and reduction of cancer cell viability in ex vivo cultured tissue slices of human PDAC. **(A)** (Top) Overview of cultured tissue slices with cell outgrowth annotations as lines in different colors corresponding to the various outgrowth types (scale bar 500 μm). (Bottom) Representative examples (H&E-staining) of the different outgrowth types and grouping for analysis (“Viable”, “Damaged”) (scale bar 50 μm). **(B)** Representative photomicrographs of a moderately differentiated PDAC in untreated (left) and treated (right) tissue slices (scale bar 50 μm).

Six moderately differentiated tumors showed a prominent cancer cell outgrowth on the surface of the tissue slices during culture. The cancerous outgrowth showed various histomorphological appearances. The “flat”, “cubic”, “cylindrical” and “clear” (mildly vacuolated) cell outgrowths were predominant in control slices and were grouped as “*viable*” cancer cell outgrowth, as they often formed an extensive and continuous cell outgrowth without overt signs of severe cell damage or cell death (**Figure 2A**).

Two further outgrowth morphologies were most prominent in slices treated with sodium selenite: a “*swollen*” type that formed a patchy and discontinuous layer of cells with enlarged, markedly rounded, and clear cytoplasm, which tended to detach from the surface of the tissue slice; and a “*necro-apoptotic*” outgrowth composed of overtly necrotic or apoptotic, fragmented, hyperchromatic cells and cell debris. Together, the “swollen” and “necro-apoptotic” cell outgrowths were grouped as “*damaged*” cancer cell outgrowth. When assessing the tumor in the center of the slices, similar cancer cell morphologies were observed. The “viable” and “damaged” cancer cell categories were finally used for quantitative analysis. Representative examples of the different cell morphologies are shown in **Figure 2A** (cell outgrowth) and **Figure 2B** (within the slice).

### Sodium selenite treatment markedly reduced PDAC cell viability in a dose-dependent manner

**Figure 3:**
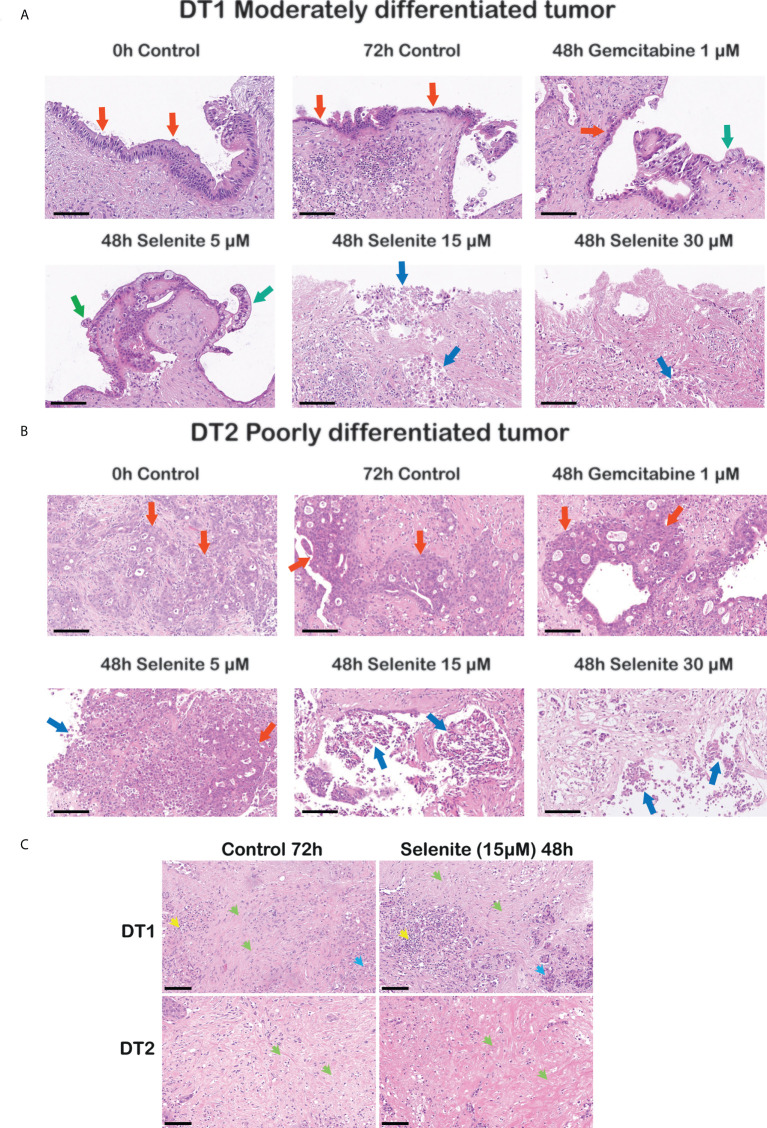
Cancer-specific cytotoxicity of sodium selenite treatment in ex vivo cultured tissue slices of human PDAC. Representative photomicrographs illustrating dose-dependent cytotoxic effect in **(A)** a moderately and **(B)** a poorly differentiated PDAC upon sodium selenite treatment at increasing concentrations. Red arrows indicate viable (cylindrical, cubic or poorly differentiated) PDAC epithelium, green arrows viable clear PDAC epithelium, and blue arrows severely damaged, necro-apoptotic PDAC epithelium. Scale bars: 100 μm. **(C)** Representative photomicrographs showing stroma preservation in untreated (Control 72h) and sodium selenite 15 µM treated slices. Green arrows indicate preserved stroma, blue arrows remnants of pancreatic parenchyma, and yellow arrows immune cells. Scale bars: 100 μm.

**Figure 4:**
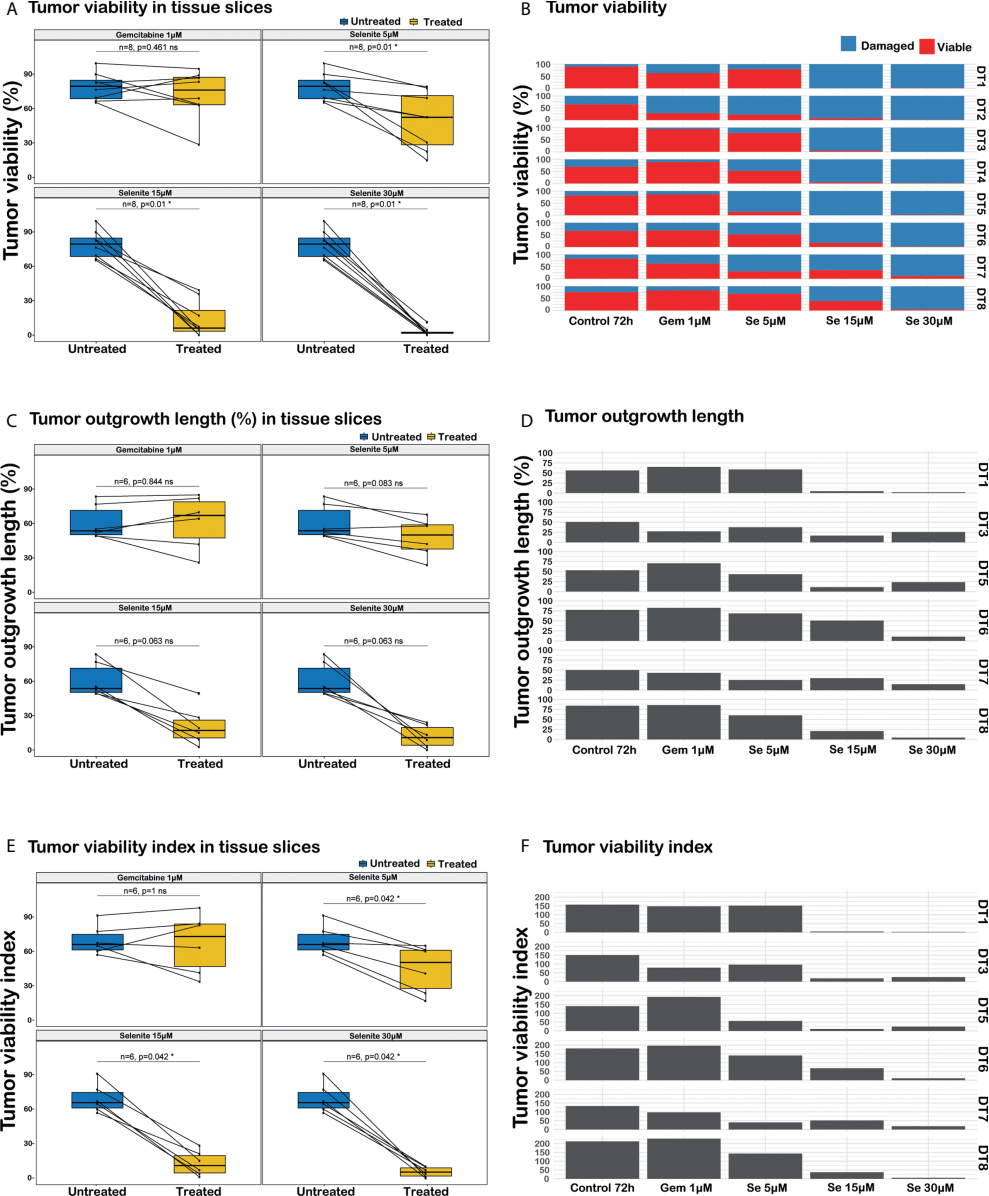
Analysis of responses to sodium selenite treatments in ex vivo cultured tissue slices of human PDAC. **(A)** PDAC cell viability, **(B)** Quantitation of PDAC cell viability in the different treatment conditions, **(C)** Cancerous outgrowth length, **(D)** Quantitation of cancerous outgrowth length in the different treatment conditions, **(E)** Tumor viability index (TVI), **(F)** Quantitation of tumor viability index in the different treatment conditions, TVI is defined as Σ_i=viable, damaged_ (% outgrowth length_i_ * weight_i_), where weight_viable_=3 and weight_damaged_=1. Statistical significance according to paired Wilcoxon signed-rank test with Benjamini-Hochberg correction for multiple testing and α-value 0.05.

Sodium selenite at 5 µM showed minimal or no cytotoxic effect, whereas sodium selenite above 15 µM showed a pronounced cytotoxic effect in all the treated samples (**Figures 3A, B**). The viability of the PDAC cells was markedly reduced with increasing doses of sodium selenite treatment (viability matrix, **Figures 4A, B**). The median percentage of viable cancer cells in control tissue was 79.4% compared to 9.3% in sodium selenite-treated tissues, all concentrations together. Major cytotoxic responses defined as over 90% of tumor cells being damaged in a sample were observed for seven and five out of eight specimens in 30 µM and 15 µM sodium selenite concentrations, respectively. These antitumoral responses correspond to tumor regression grades III-IV according to Evans regression grading system (28). Significantly reduced median viability of the PDAC cells was observed in each sodium selenite treatment concentration (i.e., 5-30 µM) as compared to untreated controls (paired Wilcoxon signed-rank test *p*-values 0.010). Moreover, the median viability was reduced in a dose-dependent manner in sodium selenite-treated tissues, ranging from 52.4% to 1.9% from 5 µM to 30 µM sodium selenite concentrations, respectively (**Figures 4A, B**; **Supplementary Table 1**). To test the significance of the dose-dependent cell viability reduction across the different treatment conditions a Friedman test was estimated showing p-value below 0.001 with an effect size of 0.847.

In summary, careful histomorphological evaluation of control and treated tissue slices demonstrated major and dose-dependent cytotoxic effects on PDAC cells in patient derived, ex vivo cultured tissue slices upon sodium selenite treatment.

### Sodium selenite treatment showed a dose-dependent decrease in total PDAC cell outgrowth length

The total length of cancer cell outgrowth varied between treatment conditions (outgrowth length matrix, **Figures 4C, D**). The median outgrowth length in control and sodium selenite-treated tissue slices, all concentrations together, was 54.7% and 24.2%, respectively. PDAC outgrowth length showed a decreasing trend upon sodium selenite treatment but did not reach statistical significance. This was reduced in sodium selenite-treated tissues, ranging its median from 51.0% to 12.6% between 5 µM and 30 µM sodium selenite concentrations (**Figures 4C, D**; **Supplementary Table 1**).

These results suggest that sodium selenite treatment may be associated with a trend towards decreased length of PDAC cell outgrowth in ex vivo cultured tissue slices, but that this readout alone may fall short to fully capture drug effect and factoring in cancer cell viability would be more relevant.

### PDAC cell outgrowth viability index was reduced in a dose-dependent manner by sodium selenite treatment

Combining in a standardized measurement the two previous readouts, a tumor viability index was defined as the summation of the products of the outgrowth lengths and viability weights, according to the formula “Σ_i=viable, damaged_ (% outgrowth length_i_ * weight_i_)”, with weight values 3 and 1 for viable and severely damaged cell outgrowth, respectively. The median PDAC cell outgrowth viability index in control and sodium selenite treated samples, all concentrations together, was 154.0 and 31.1, respectively. This was significantly reduced in each sodium selenite treatment concentration (i.e., 5-30 µM) as compared to untreated controls (paired Wilcoxon signed-rank test *p*-values 0.042, **Figures 4E, F**). Furthermore, the PDAC cell outgrowth viability index was reduced in a dose-dependent manner in sodium selenite-treated tissues, ranging from 118.0 to 14.4 from 5 µM to 30 µM sodium selenite concentrations, respectively (**Figures 4E, F**; **Supplementary Table 1**). To test the significance of the dose-dependent tumor viability index reduction across the different treatment conditions a Friedman test was estimated showing p-value below 0.001 with an effect size 0.778.

The combined evaluation of median PDAC outgrowth length together with its viability (i.e., tumor viability index) also showed a significant and dose-dependent effect of sodium selenite treatment. This was consistent with the single readouts above and suggests that combining cancer outgrowth length and viability status may allow for a comprehensive, robust and reliable assessment of drug effect in ex vivo treated tissue slices of human PDAC.

### Tissue slice stroma was detected without sign of sodium selenite treatment-related stroma cell damage

Although not quantified, qualitative assessment based on the histomorphological evaluation of H&E stained sections from both treated and untreated slices on 72h showed that the stroma, which is characteristically abundant in PDAC, was consistently detected. Both the extracellular eosinophilic stroma matrix and the variable number of mesenchymal cells, mostly identifiable by their elongated nuclei, were present in the slices’ stroma. We did not detect obvious signs of treatment-related stroma cell damage as observed in the cancer cells, i.e., there was no sign of obvious apoptosis/necrosis in the stromal cells. Representative photomicrographs of the stroma in untreated (control 72h) and Se15 µM treated slices are shown in **Figure 3C**.

### Gene expression analysis identified downregulation of PDAC aggressiveness genes and upregulation of cell death genes upon sodium selenite treatment

To identify the genes affected by sodium selenite (15 µM) treatment, differential gene expression analysis showed that 1099 genes (out of 53594, 2.1%) were overexpressed and 738 (1.4%) were underexpressed in sodium selenite treated slices as compared to cultured, untreated slices.

**Figure 5:**
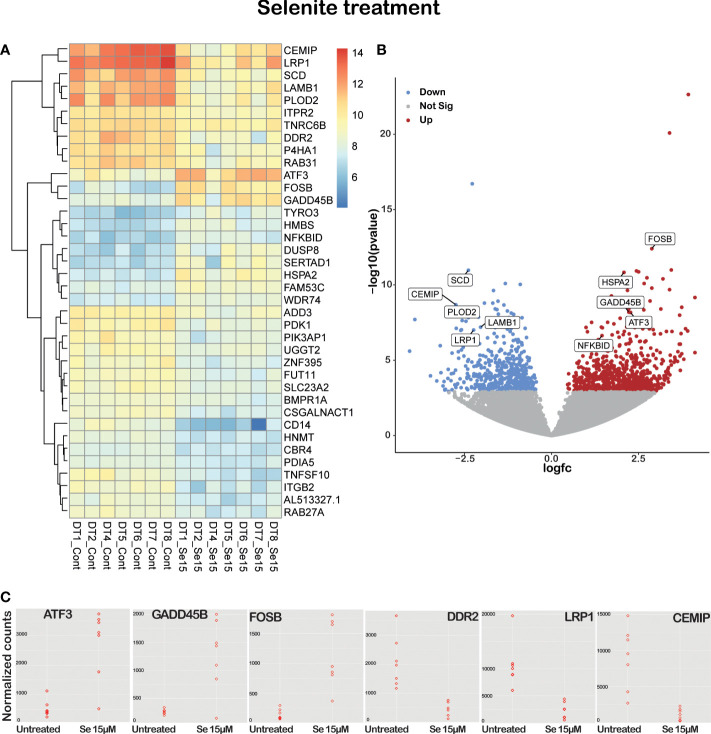
Sodium selenite targeting genes in *ex vivo* cultured tissue slices of human PDAC. **(A)** Supervised hierarchical clustering with RNA expression heatmap. **(B)** Volcano plot of significant differentially expressed/labeled genes between untreated and 15 µM sodium selenite treated tissue slices. **(C)** Dot plot of several relevant differentially expressed genes between untreated and 15 µM sodium selenite treated samples (n=7).

To evaluate the most significant differentially expressed genes, results were filtered by restricting the p-value cutpoint to 0.0001 followed by exclusion of transcripts with very low alignment counts, i.e., less than 350 reads. This resulted in a list of 38 differentially expressed genes (**Figures 5A–C**, clustered RNA expression heatmap). Specifically, CEMIP, PLOD2, DDR2 and P4HA1, genes involved in cancer growth, extracellular matrix remodeling, and metastatic potential, were significantly downregulated, while the cell death-inducing genes ATF3 and ACHE were significantly upregulated in the tissue slices treated with Se15 µM as compared to untreated controls (**Supplementary Table 2**).

Sodium selenite treatment upregulated pro-apoptotic, apoptotic, and tumor suppressor genes such as ATF3, FOSB, GADD45B, DUSP8 and ACHE, and downregulated tumor cell survival genes such as SCD, LRP1, RAB31, SRPX2, PDK1 and CSGALNACT1. It has been shown by several groups that selenium can sensitize drug-resistant cancer cell lines and induce apoptosis. In our study, sodium selenite treatment downregulated chemoresistance genes such as PDK1, PIK3AP1 and P4HA1. Tumor-promoting inflammation is another important hallmark of cancer that sodium selenite treatment reduced by downregulating CD14 and ZNF395 genes (**Supplementary Table 2**).

On pathways analysis, 764 genes were over-represented in 1568 suggested pathways. The top three and most significant associated pathways were the ‘Attenuation phase of the heat shock transcriptional response (Pathway ID: R-HSA-3371568)’, ‘Collagen formation (Pathway ID: R-HSA-1474290)’ and ‘Heat shock factor protein 1 (HSF1)-dependent transactivation (Pathway ID: R-HSA-3371571)’.

One well-established mechanism by which moderate to high doses of sodium selenite exert cytotoxic effects is through the massive induction of ROS due to redox cycles with thiols in the presence of oxygen (13). Because of the resistant phenotype expressed by tumor cells, these cells will be much more sensitive and the redox cycles will be more pronounced due to induced thiol levels. Although the detailed investigation of the mechanisms underpinning the cytotoxic effect of sodium selenite treatment on PDAC are beyond the scope of this study, the detected downregulation of cancer-cell aggressivess and upregulation of cell-death related genes supports from a transcriptional level the cancer cell damage detected on histomorphology in sodium selenite treated tissue slices. Pathway overrepresentation of heat shock response, which is known to be triggered in response to oxidative stress (29), is also well consistent with high-dose sodium selenite treatment, which induces the production of high levels of reactive oxygen species (ROS) selectively in the cancer cells, as previously reported by our group (11).

### Gemcitabine 1 µM treatment lacked effect in *ex vivo* cultured PDAC tissue slices

PDAC tissue slices treated with Gem 1 µM only showed a notable decrease in PDAC cell viability in one (DT2) out of 8 tumors. For Gem 1 µM treated slices, the median cancer cell viability, outgrowh length, and viability index were 76.1% (vs. 79.4% in untreated controls), 67.8% (vs. 54.7%), and 170.0 (vs. 154.0), respectively (**Figure 4**; **Supplementary Table 1**). Overall, the differences between untreated and Gem 1 µM treated slices were not statistically significant (paired Wilcoxon signed-rank test *p*-values range between 0.461 and 1).

## Discussion

Our most important finding from this study is that sodium selenite showed a pronounced and significant cytotoxic effect on PDAC without any damage to non-malignant tissue components in the *ex vivo* organotypic slice culture model. To our knowledge, this effect of sodium selenite on PDAC is superior to any other drug tested, and thus of great clinical relevance. Moreover, the concentrations used for tumor-specific cell death were well below the MTD for cancer patients (15).

PDAC is a devastating disease with an alarming increase in incidence (1). The currently available chemotherapeutic regimens are associated with pronounced drug resistance, underlining the urgent need for novel treatment approaches. One challenge in developing an efficient drug to treat pancreatic cancer is to make the compound accessible to the tumor cells surrounded by desmoplastic stroma.

During the past decade, a growing body of literature has used *ex vivo* slice culture models to address key questions related to oncogenic signaling pathways, drug sensitivity testing, and immunotherapy in different tumor types (30–33). In the present study, we used an *ex vivo* organotypic pancreatic cancer slice culture model to evaluate the effect of sodium selenite. The main advantage of our *ex vivo* model compared to traditional cell culture (2D, 3D, organoids), xenograft-based, or genetically engineered mouse models is the preservation of the native tumor microenvironment and the 3D-tissue architecture, which makes our model an ideal surrogate for the *in vivo* tumor.

However, a note of caution is that the choice of readouts for evaluating drug response-associated tumor cell damage is of paramount importance and is yet to be broadly established for studies based on tissue slice cultures. Indeed, previous studies have used quantitative readouts of drug effects including differences between matched treated and untreated slices in total tumor cell content (31, 34), tumor cell proliferation, cell death, and functional assays of cell viability (34). While these methods may be suitable for cancer types that form large, compact masses of tumor cells, PDAC features limit their application. In contrast to most other cancer types, PDAC is characterized by the presence of a prominent stroma, a lower tumor cell density, and a more dispersed tumor growth (35). PDAC cells are also prone to intermix with non-tumorous pancreatic remnants, which impairs their explicit discrimination and may require a panel of immunohistochemical markers for their precise identification (36). Besides, the time lapse from drug exposure to slice harvesting must be considered, as certain proteins and transcripts may degrade and become undetectable in advanced phases of cell death, as well as the particular action mechanisms of the investigated drugs. During preliminary, explorative investigations (data not shown), we noticed that morphologically damaged PDAC cells continue proliferating, possibly reflecting a stress response to the cytotoxic effects of selenium, which are known to be mediated by other mechanisms than cytostasis. While apoptotic markers (activated caspase 3 and M30) could no longer be detected in a fraction of severely damaged, nearly necrotic cancer cells, likely due to protein degradation at an advanced phase of cell death (data not shown). Hence, we concluded that a precise assessment of cancer cell morphology was the most reliable and consistent readout for the evaluation of drug response-associated cell damage in precision-cut tissue slices of human PDAC after sodium selenite treatment. This was preferably performed in the cancerous cell outgrowth (in 6 out of 8 cultures) rather than within the slice (in 2 cultures), where PDAC cells frequently intermix with non-tumorous pancreatic remnants, and patchy areas of culture-related tissue damage may occur, which could be difficult to differentiate from drug effect. We herein report that sodium selenite showed a pronounced and significant cytotoxic effect on PDAC without any damage to non-malignant tissue components in the *ex vivo* organotypic slice culture model. The concentrations used for tumor-specific cell death were well below the MTD in cancer patients (15) and therefore highly likely to be of great clinical relevance. Thus, the results are very promising and to our knowledge, the effect of sodium selenite is superior to any other tested drug or treatment regimen published.

Selenium is an essential micronutrient that acts both as an antioxidant and at higher doses as a pro-oxidant. Its biological effects strictly depend on chemical speciation, applied dose, and exposure duration. The window of requirement and toxicity is relatively narrow for selenium (37, 38). Our group and others have shown that selenium compounds induce tumor-specific cytotoxicity, especially in highly resistant cancer cells at a concentration that does not affect normal, non-malignant cells (9, 10).

Our data presented herein show remarkably prominent cytotoxic effects with sodium selenite in the *ex vivo* model. Sodium selenite at 5 µM had a minimal or no cytotoxic effect, whereas sodium selenite above 15 µM showed a pronounced cytotoxic effect (**Figures 3A, B**). We previously described that culturing conditions alone have a limited impact on untreated organotypic PDAC (39). In the present study, transcriptomic data revealed that 15 µM of sodium selenite targeted hallmark genes that support cancer progression, with significant downregulation of genes (CEMIP, PLOD2, DDR2, and P4HA1) associated with cancer cell survival, extracellular matrix remodeling, and metastatic potential, together with upregulation of cell death associated genes (ATF3 and ACHE) (40). Altogether, the presented transcriptomic data supports the potential of selenium compounds in targeting multiple pathways described as hallmarks of cancer (41). PDAC is known to be metabolically vulnerable and the tumor stroma has been suggested to play a crucial role for the growth and survival of PDAC cells (42). In particular, the cysteine/thiol levels, which are crucial for growth and viability of PDAC cells, will be decreased or abolished by sodium selenite treatment. The transcriptional changes reported herein are thus to be considered as a normalization of the increased expression in the cancer cells of tumor growth promoting genes, triggered by the excess ROS produced by high-dose sodium selenite. Thus, the effects of the extracellular matrix modulating genes are likely to be a contributing factor to the tumor specific cytotoxicity clearly showed herein. We have recently published the first phase I clinical trial of intravenous (*iv)* sodium selenite in patients with end-stage cancers of various origins (15). The tolerance to sodium selenite was remarkably high with an estimated maximum tolerated dose of 10.2 mg (Se)/square meter body surface. Even though the study was a phase I study with safety as the primary end-point we also noted clinical effects. We observed a very short half-life of sodium selenite and therefore we now perform a modified phase I trial with continuous administration of sodium selenite iv. This phase I clinical trial shows that even a therapeutically relevant concentration of 30 µM sodium selenite can be physiologically tolerable for benign cells.

In conclusion, our data reveal that sodium selenite is a promising candidate for the treatment of pancreatic ductal adenocarcinoma. The effects were pronounced and strikingly tumor-specific, with preserved non-malignant tissue structures, and the applied concentrations were well below the MTD for humans. A strength of the study is also the consistent results in tissues from eight different individual patients. To our knowledge effects of this magnitude and reproducibility among different patients with PDAC have previously not been reported for any treatment regimen.

## Data availability statement

The RNA-Seq data presented in the study are deposited in the European Nucleotide Archive (ENA) database (https://www.ebi.ac.uk/ena), accession numbers ERR10030082 - ERR10030095.

## Ethics statement

The studies involving human participants were reviewed and approved by the Regional Ethical Review Board, Stockholm/Etikprövningsmyndigheten (decision numbers 2012/1657-31/4, 2018-2654/32 and 2019-00788). The patients/participants provided their written informed consent to participate in this study.

## Author contributions

MB, CM and AS conceived the idea of the study. AS performed all the *ex vivo* culturing. SE performed tissue embedding, sectioning, and H&E staining; CM evaluated, annotated, and generated all the results from the H&E staining and analyzed the data quantitatively together with MGe and VP. BB and CM performed immunohistochemical assessments. MGh performed the transcriptomic analysis; MB, CM, AS, VP, JD and MGh performed all the data analysis. MB, CM, AS and MGh wrote the manuscript. JD, VP, MGe and BB commented on the manuscript. MGe and BB commented on the manuscript. All authors reviewed the final version of the manuscript.

## Funding

This study was supported by grants from Cancerfonden (180429 and 201100PjF01H), Cancer och Allergifonden (206) and Radiumhemmets forskningsfonder (171023 and 201063) to MB. The study was also supported by the Pathology Core Facility, Karolinska Institutet/Region Stockholm.

## Conflict of interest

MB is listed as an inventor in a patent application for *i.v.* use of inorganic selenium in cancer patients and holds shares in SELEQ OY, a company involved in the development of Se-based formulations for prevention and treatment.

The remaining authors declare that the research was conducted in the absence of any commercial or financial relationships that could be construed as a potential conflict of interest.

## Publisher’s note

All claims expressed in this article are solely those of the authors and do not necessarily represent those of their affiliated organizations, or those of the publisher, the editors and the reviewers. Any product that may be evaluated in this article, or claim that may be made by its manufacturer, is not guaranteed or endorsed by the publisher.
