# Supplementary material for: Drug-induced tumor-specific cytotoxicity in a whole tissue ex vivo model of human pancreatic ductal adenocarcinoma
Source: Front Oncol. 2022 Aug 18;12:965182. doi: 10.3389/fonc.2022.965182 (PMC9436406; doi:10.3389/fonc.2022.965182)
Supplement: Supplementary file 1 [file Table_1.pdf]

Supplementary information

Supplementary Table 1: Median estimated for the tumor cell viability, tumor outgrowth length and tumor outgrowth viability index in the different treatment conditions.

Treatment condition	Tumor cell viability (%)(IQR)	Tumor outgrowth length (%)(IQR)	Tumor outgrowth viability index (IQR)
Control 72h	79.4 (16)	54.7(26.9)	154.0(31.2)
Gem 1 µM	76.1(23.9)	67.8(39.5)	170.0(85.5)
Se 5 µM	52.4 (42.9)	51 (22.7)	118.0 (76.6)
Se 15 µM	6.1 (18.2)	18.6 (18.9)	27.5 (34.7)
Se 30 µM	1.9 (1.1)	12.6 (18.9)	14.4 (16.4)

Supplementary Table 2: Functions of genes that were altered by sodium selenite treatment.

Gene ID (Log 2-fc)	Function of the protein	References
CEMIP (-2.75)	Increased expression degrades hyaluronan (HA) into small fragments which promote metastasis, cell survival, tumor progression, ECM remodeling, invasion, migration and promotes angiogenesis. Upregulation correlates with a poor prognosis. It also regulates β-catenin. Downregulation reduces the migration of cancer cells, EMT, β-catenin (reduces fibrosis) and proliferation.	[1, 2]
LRP1 (-2.24)	Promotes tumor migration, progression, invasion and metastasis by increasing MMP2 and MMP9 through the ERK pathway. Increases angiogenesis and inhibits apoptosis by regulating insulin receptors. Overexpression of this gene correlates with lymph-node invasion. It is a P53 target gene. Downregulation reduces cancer cell progression, metastasis and induce apoptosis.	[3]
SCD (-2.39)	It promotes tumor progression by increasing monounsaturated fatty acid (MUFA) level to maintain redox imbalance, suffice cancer cell energy needs and activates UPR in ER. Downregulation results in tumor growth inhibition, reduce progression, reduced MUFA level, phospholipid synthesis and induces apoptosis.	[4-6]
LAMB1 (-2.04)	Poor prognosis in various cancer, induce cell proliferation, mortality, migration, and invasion of the tumor via the ERK/c-Jun pathway. It activates MMP2 and regulates ECM remodeling. Downregulation results in reduced tumor cell proliferation, mortality, migration, metastasis and invasion.	[7]
PLOD2 (-2.39)	Induced by hypoxia (HIF-1α) and involved in ECM-fiber remodeling in the stroma of PDAC thereby increases the cancer cell morality. Promotes EMT and cancer progression. It correlates with poor prognosis in various cancer types. PLODs highly expressed in cancer-associated fibroblasts (CAFs). Downregulation block the desmoplasia (fiber remodeling) in the stroma, collagen deposition thereby reducing tumor migration and invasion. It also suppresses tumor metastasis and reduces CAFs.	[8, 9]
DDR2 (-2.1)	It is activated by fibrillar collagens and is largely expressed in mesenchymal cells. It helps in cancer progression by inducing tumor cell-collagen interactions via Akt-mediated signaling pathways. Downregulation reduces tumor growth, invasion, fibrillar collagen and metastasis.	[10]
P4HA1 (-1.65)	Induced by hypoxic (HIF-1α) condition and it involved in HIF-1α stability to promote tumor progression. It promotes metastasis, chemoresistance, glycolysis and regulates ECM remodeling (collagen). Upregulation correlates with a poor prognosis. Downregulation of this gene inhibiting tumor cell proliferation, reduce expression of stemness markers, sensitizes cells to chemotherapy and suppressed the glycolytic activity of PDAC cells.	[11]

RAB26 (2.5)	Loss of MIST1 function largely results in cells susceptible to pancreatitis. MIST1 upregulates RAB26 to regulate lysosome coalescence and redistributing mitochondria into neighborhood cells.	[12, 13]
ATF3 (2.27)	It is an adaptive response gene, which is a master regulator in metabolic and immune homeostasis. It impedes migration, invasion, induces inflammation and apoptosis by stabilizing pro-apoptotic genes such as STAT1 and Bax. It also downregulates MMP1 transcription in primary monocytes and macrophages. ATF3 can downregulate SCD1 and proinflammatory cytokines such as IL6 and IL-12b.Downregulation results in increased tumor initiation, progression, and remission because of imbalanced metabolic homeostasis. Increased proinflammatory cytokines such as IL6 and IL-12p40 following TLR activation. Suppress pro-apoptotic genes such as Bax and Bak.	[14]
FOSB (2.88)	Suppress cancer cell proliferation, migration, and clone formation. Prevent lymph node metastasis in PDAC. Reduced expression correlate with poor differentiation, lymph node metastasis and advanced TNM stage, poor prognosis, and patient survival. Induce proliferation, malignant transformation, migration and acquiring malignant potential in PDAC.	[15, 16]
GADD45B (2.22)	It has a Pro-apoptotic function which is involved in DNA damage repair, cell cycle arrest and normal cell survival. It increases phosphorylation of MAP2K3 and active P38 and P53. It plays a role in cell cycle checkpoint by inhibiting cdc2/cyclinB1 kinase and inhibit metastasis. Downregulation promotes metastasis and tumor cell progression.	[5, 17]
PDK1 (-1.67)	It can phosphorylate several kinases pathways to induced tumorigenesis such as PI3K, Akt, SGK3, etc. It is important for T-cell receptor signaling to convey its signal for maturation and migration of endothelial cells, neutrophil chemotaxis and angiogenesis. Downregulation results in reduced cancer progression, invasion, migration, metastasis, angiogenesis, anchorage-dependent and anchorage-independent growth in PDAC and several other cancer types. It sensitizes the tumor cells to chemotherapy.	[18, 19]
PIK3AP1 (-1.62)	It is the upstream regulator of the PI3K/Akt/c-Myc pathway. It induces tumor progression and chemoresistance. It is important for tumor cell proliferation, metabolism and survival. Downregulation inhibits tumor progression and sensitize the cells to chemotherapy.	[20]
ZNF395 (-1.53)	It is induced by hypoxic conditions and increases the survival of PDAC. It promotes inflammation-mediated cancer progression via NFkB and HIF pathways. It induces IFNα stimulating genes. Downregulation reduces IFNα-mediated stimulating of innate immune response and reduces cancer cell progression.	[21, 22]
CSGALNACT1 (-1.33)	It promotes adhesion, invasion, metastasis, migration, EMT, angiogenesis, apoptosis, and ECM remodeling by increasing heparan sulfate (HS) and chondroitin sulfate (CS). It is also involved in hyaluronan synthesis which promotes tumor proliferation and migration. Downregulation of this gene inhibit metastasis, cell proliferation, EMT, invasion and migration	[23]
CD14 (-2.57)	In tumor cells with high CD14 is important in TLRs signaling pathways which stimulates tumor microenvironment for proliferation and progression. It also helps in IL6 production and is highly vascularized with myeloid cell infiltration and retention. It produces inflammatory factors that activate angiogenesis to maintain an immune-suppressive environment for tumor cells.	[24]
RAB31 (-1.57)	Increased RAB31 expression induces uncontrolled proliferation, tumor development, migration, and metastasis through ERK1/2 and PI3K/Akt pathways. Poor prognosis for PDAC patients.	[25]
SRPX2 (-1.82)	Considered an oncogene that promotes proliferation, migration and invasion of cancer cells. Poor prognosis for PDAC patients.	[25]

ACHE (1.72)	A low level of ACHE revealed a poor prognosis in HCC. ACh (acetylcholine) hyper-activate nAChRs (nicotinic acetylcholine receptor) to induce angiogenesis, migration, and proliferation of tumor cells. Increased expression induces apoptosis and inhibits cancer proliferative pathways such as PI3K/Akt (cancer stem cell maintaining pathway). Higher expression showed longer survival in patients. ACh level is regulated by AChE protein.	[26]
MAFA (3.39)	Downregulated in various cancer type. Expressed in β-cells and it is the positive indicator of health and function. It activates insulin transcription in high glucose conditions.	[27]
DUSP8 (1.97)	Attenuate the ERK/Akt pathway thereby reducing the tumor proliferation and migration in vivo. DUSP controls the MAPK pathway. DUSP8 might be a tumor suppressor gene.	[28, 29]

References

1. Koga A, Sato N, Kohi S, et al. KIAA1199/CEMIP/HYBID overexpression predicts poor prognosis in pancreatic ductal adenocarcinoma. Pancreatology 2017;17:115-122.
2. Deroyer C, Charlier E, Neuville S, et al. CEMIP (KIAA1199) induces a fibrosis-like process in osteoarthritic chondrocytes. Cell Death Dis 2019;10:103.
3. Gheysarzadeh A, Ansari A, Emami MH, et al. Over-expression of low-density lipoprotein receptor-related Protein-1 is associated with poor prognosis and invasion in pancreatic ductal adenocarcinoma. Pancreatology 2019;19:429-435.
4. Tracz-Gaszewska Z, Dobrzyn P. Stearoyl-CoA Desaturase 1 as a Therapeutic Target for the Treatment of Cancer. Cancers (Basel) 2019;11.
5. Han S, Wang Y, Ma J, et al. Sulforaphene inhibits esophageal cancer progression via suppressing SCD and CDH3 expression, and activating the GADD45B-MAP2K3-p38-p53 feedback loop. Cell Death Dis 2020;11:713.
6. Wu XY, Zou XJ, Chang Q, et al. The Evolutionary Pattern and the Regulation of Stearoyl-CoA Desaturase Genes. Biomed Research International 2013;2013.
7. Lee H, Kim WJ, Kang HG, et al. Upregulation of LAMB1 via ERK/c-Jun Axis Promotes Gastric Cancer Growth and Motility. Int J Mol Sci 2021;22.
8. Qi Y, Xu R. Roles of PLODs in Collagen Synthesis and Cancer Progression. Front Cell Dev Biol 2018;6:66.
9. Sada M, Ohuchida K, Horioka K, et al. Hypoxic stellate cells of pancreatic cancer stroma regulate extracellular matrix fiber organization and cancer cell motility. Cancer Lett 2016;372:210-8.
10. Wasinski B, Sohail A, Bonfil RD, et al. Discoidin Domain Receptors, DDR1b and DDR2, Promote Tumour Growth within Collagen but DDR1b Suppresses Experimental Lung Metastasis in HT1080 Xenografts. Sci Rep 2020;10:2309.
11. Cao XP, Cao Y, Li WJ, et al. P4HA1/HIF1alpha feedback loop drives the glycolytic and malignant phenotypes of pancreatic cancer. Biochem Biophys Res Commun 2019;516:606-612.
12. Jin RU, Mills JC. RAB26 coordinates lysosome traffic and mitochondrial localization. Journal of Cell Science 2014;127:1018-1032.
13. Tian X, Jin RU, Bredemeyer AJ, et al. RAB26 and RAB3D are direct transcriptional targets of MIST1 that regulate exocrine granule maturation. Mol Cell Biol 2010;30:1269-84.
14. Ku HC, Cheng CF. Master Regulator Activating Transcription Factor 3 (ATF3) in Metabolic Homeostasis and Cancer. Front Endocrinol (Lausanne) 2020;11:556.
15. Kim JH, Lee JY, Lee KT, et al. RGS16 and FosB underexpressed in pancreatic cancer with lymph node metastasis promote tumor progression. Tumour Biol 2010;31:541-8.
16. Tang C, Jiang YS, Shao WW, et al. Abnormal expression of FOSB correlates with tumor progression and poor survival in patients with gastric cancer. International Journal of Oncology 2016;49:1489-1496.
17. Vairapandi M, Balliet AG, Hoffman B, et al. GADD45b and GADD45g are cdc2/cyclinB1 kinase inhibitors with a role in S and G2/M cell cycle checkpoints induced by genotoxic stress. Journal of Cellular Physiology 2002;192:327-338.
18. Emmanouilidi A, Falasca M. Targeting PDK1 for Chemosensitization of Cancer Cells. Cancers (Basel) 2017;9.

19. Emmanouilidi A, Fyffe CA, Ferro R, et al. Preclinical validation of 3-phosphoinositide-dependent protein kinase 1 inhibition in pancreatic cancer. J Exp Clin Cancer Res 2019;38:191.
20. Zhang F, Li K, Yao X, et al. A miR-567-PIK3AP1-PI3K/AKT-c-Myc feedback loop regulates tumour growth and chemoresistance in gastric cancer. EBioMedicine 2019;44:311-321.
21. Gong HJ, Wu TT, Clarke EM. Pathway-gene identification for pancreatic cancer survival via doubly regularized Cox regression. Bmc Systems Biology 2014;8.
22. Jordanovski D, Herwartz C, Pawlowski A, et al. The hypoxia-inducible transcription factor ZNF395 is controlled by IkB kinase-signaling and activates genes involved in the innate immune response and cancer. PLoS One 2013;8:e74911.
23. Afratis N, Gialeli C, Nikitovic D, et al. Glycosaminoglycans: key players in cancer cell biology and treatment. FEBS J 2012;279:1177-97.
24. Cheah MT, Chen JY, Sahoo D, et al. CD14-expressing cancer cells establish the inflammatory and proliferative tumor microenvironment in bladder cancer. Proc Natl Acad Sci U S A 2015;112:4725-30.
25. Li H, Zhang SR, Xu HX, et al. SRPX2 and RAB31 are effective prognostic biomarkers in pancreatic cancer. J Cancer 2019;10:2670-2678.
26. Xu H, Shen Z, Xiao J, et al. Acetylcholinesterase overexpression mediated by oncolytic adenovirus exhibited potent anti-tumor effect. BMC Cancer 2014;14:668.
27. Aramata S, Han SI, Kataoka K. Roles and regulation of transcription factor MafA in islet beta-cells. Endocr J 2007;54:659-66.
28. Ding T, Zhou Y, Long R, et al. DUSP8 phosphatase: structure, functions, expression regulation and the role in human diseases. Cell Biosci 2019;9:70.
29. Ding T, Cui PP, Zhou Y, et al. Antisense Oligonucleotides against miR-21 Inhibit the Growth and Metastasis of Colorectal Carcinoma via the DUSP8 Pathway. Molecular Therapy-Nucleic Acids 2018;13:244-255.
